# A New Stress-Released Structure to Improve the Temperature Stability of the Butterfly Vibratory Gyroscope

**DOI:** 10.3390/mi10020082

**Published:** 2019-01-24

**Authors:** Fenlan Ou, Zhanqiang Hou, Tongqiao Miao, Dingbang Xiao, Xuezhong Wu

**Affiliations:** College of Intelligence Science and Engineering, National University of Defense Technology, Changsha 410073, China; oufenlan15@nudt.edu.cn (F.O.); miaotongqiao12@nudt.edu.cn (T.M.); dingbangxiao@nudt.edu.cn (D.X.); xzwu@nudt.edu.cn (X.W.)

**Keywords:** stress-released structure, frequency mismatch, temperature stability

## Abstract

This paper is devoted to discussing the influence of thermal stress on the performance of the Butterfly Vibratory Gyroscope (BFVG). In many gyroscopes, due to the material properties and the fabrication processes, the deformation caused by residual stress or thermal mechanical stress is of great concern since it directly affects the performance. Here, a new stress-released structure was proposed to reduce the deformation to improve BFVG’s performance considering the symmetry of the electrode and the miniaturization of the structure. Its dimensional parameters relate to the effect of thermal stress release and the stiffness characteristics of the BFVG’s oblique beam. The single parameter analysis method was used to explore the influence of the parameters on the effect of thermal stress release to guide the optimal size of the final design. The effect of thermal stress release in the BFVG at the full range temperature was also tested after the fabrication. The results showed that the influence of thermal stress on the BFVG’s performance effectively reduced.

## 1. Introduction

Micro-machined vibratory gyroscopes are important components in inertial navigation systems, which are widely used in both military and civil fields [1,2]. Their major advantages include miniaturization, batch fabrication, low cost, high reliability, and IC compatible integration [3]. In the market of tactical and inertial grade gyroscopes, proving the reliability and long-term stability of these devices remains probably the greatest challenge. The parallel plate electrodes gyroscope is a typical micro-machined vibratory gyroscope. The Butterfly vibratory gyroscope (BFVG) is classified as one kind of the high sensitivity parallel plate electrodes gyroscope due to its unique structure, and has been widely used and studied [4,5,6]. Compared with other gyroscopes, the BFVG has superiority in size, accuracy, operation life, and performance.

The basic architecture of the BFVG comprises a drive mode and a sense mode. The drive mode generates and maintains a constant linear or angular momentum, and the sense mode measures the Coriolis force induced by drive vibration and angular rate input. The motion pattern of the drive mode of the BFVG is reflected in the flexural vibration of the oblique beam, which is easily affected by the residual stress or thermal mechanical stress. Due to non-ideal factors, such as manufacturing imperfections and temperature changes, the key parameters of the sensitive structure of the gyroscope will deviate from the ideal design values, and the stability of parameters will significantly impact on the final performance of the gyroscope. The deformation caused by residual stress or thermal mechanical stress is an important problem, because it not only affects the performance [7,8,9], but also causes frequency change [10,11,12], rigid axis deflection, bias, scale factor drift [13,14,15], even breakage of the structure [16]. The deviation of the operating frequency due to pulling stress is a critical issue, and it should be controlled or eliminated to avoid influencing the gyroscope’s performance [17]. In recent years, the researchers have gradually shifted the research focus from the new principle design to the stability of the gyroscope. Numerous studies have illustrated the optimization design of the sensing structure of micro-electromechanical systems (MEMS) gyroscopes [18]. To achieve the gyroscope’s stability, the mechanical part was designed with a one-sided open frame to mitigate the variation of resonant frequencies caused by thermal stress in [19]. Thermal stress structures with holes and grooves are fabricated at the bottom of the resonator which can generate thermal stress to make the vibrating axis rotate in the opposite direction to reduce the gyroscope’s temperature drift [20]. Chungbuk National University proposed a semi-folded spring to reduce the effect of the structural stress on the working state. This method effectively reduces the frequency shift [16]. The Silicon Sensing Company of the United Kingdom adopted a Deep-reactive ion etching fabrication process to design a gyroscope. Because of the bracing form of the Z-shaped bending beam, the change of the initial gap with ambient temperature has been effectively avoided [21,22,23]. The Georgia Institute of Technology has improved the anchor support pattern of the sensing structure on the basis model of the vibratory ring gyroscope. This structure is supported by a single anchor point, which has decreased the stress and the deviation of the operating frequency [24]. Tsinghua University proposed a center-supported four-mass block vibratory gyroscope, and it uses a special combination of Y-beam and N-beam to support itself. This method effectively releases the internal stress of the structure [25]. In the above aspects, the optimal design of the stress-released structure is the most convenient and effective method to improve the BFVG, the Sensonor (Sensonor is a Norwegian company specializing in the development and production of high-precision gyroscopes.) has etched holes through the mass in order to decrease the damping effects, and increase the Q-factors [26]. This design causes the asymmetry of the electrode and mass which can affect the performance of the gyroscope. The National University of Defense Technology has designed an elastic stress-released frame structure to reduce the heat stress, and improve the performance and stability of the gyroscope [27]. However, this design can’t miniaturize the structure of the gyroscope. Based on the above researches, the purpose of this paper is to design a new stress-released structure that will not affect the symmetry of the gyroscope and increase the size of the gyroscope.

In this paper, a new stress-released structure will be proposed to reduce the effect of thermal stress to improve BFVG’s performance. In the Section 1, the research background is introduced. In the Section 2, the theoretical foundations of thermal stress will be analyzed. In the Section 3, the dimensional parameters of the stress-released structure are related to the effect of thermal stress release and the stiffness characteristics of the gyroscope’s oblique beam. Therefore, the single parameter analysis method is used to simulate the temperature characteristics of the operating frequencies and the frequency mismatch of the BFVG in the whole temperature range. The influence of the parameters on the effect of thermal stress release is explored to guide the optimal size of the final design. In the Section 4, the performance of the gyroscope will be tested after the fabrication processes. In conclusion, the performance test confirmed that the new stress-released structure will reduce the effect of thermal stress on the gyroscope’s performance. 

## 2. Thermal Stress Analysis

### 2.1. The Theoretical Principle of the Butterfly Vibratory Gyroscope (BFVG)

**Figure 1:**
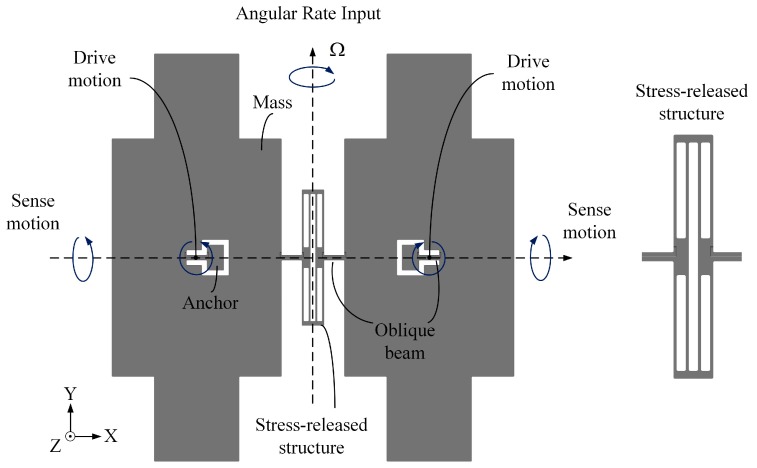
Sensing structure of the Butterfly vibratory gyroscope (BFVG).

Figure 1 shows the vertical-view of the developed sensing structure of the BFVG used in this paper. The sensing structure includes four proof-masses (two masses are connected, they are symmetrically distributed at the anchor point), two cantilever beams, one stress-released structure, one oblique beam, and two anchors. There is an electrode gap between the sensing structure and the electrode structure after bonding.

**Figure 2:**
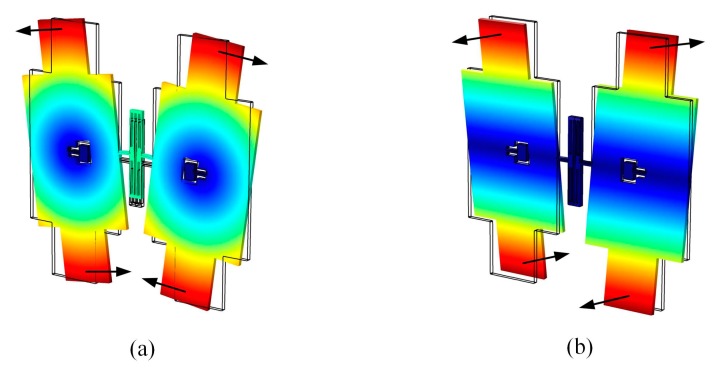
The operating modes of the BFVG: (**a**) The drive mode; (**b**) the sense mode.

The BFVG has two orthogonal operation modes: drive mode and sense mode. The flexural vibration of the oblique beam will cause the movement of the proof-masses in the tangential direction of the structural plane (drive mode), as shown in Figure 2a. The torsional vibration of the oblique beam will cause the movement of the proof-mass in the normal direction of the structural plane (sense mode), as shown in Figure 2b. The sensing structure of the BFVG operates at the resonant frequency of the drive mode under the excitation of the externally driving force. The four proof-masses move periodically at a constant frequency and amplitude. When there is an angular rate, the Coriolis force will couple the drive mode to the sense mode. The input angular velocity can be obtained by measuring the displacement of the coupled vibration [6].

**Figure 3:**
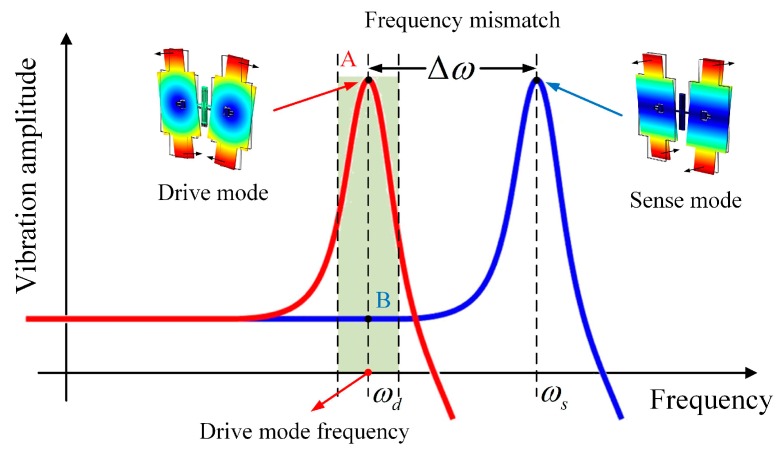
The frequency mismatch about the drive mode and the sense mode of the BFVG.

According to the operating principle, the BFVG can be modeled as a mass-spring-damper system. For the BFVG, the resonant frequency of the sense mode is greater than that of the drive mode. It operates in the frequency mismatching mode (Figure 3). The frequencies of the drive and sense mode are $\omega_{d}$ and $\omega_{s}$ respectively. Δω is the difference between the operating modes. It is one of the most important parameters that affect the performance of the BFVG.

### 2.2. Thermal Stress of the BFVG

The resonance frequency of the BFVG has been analyzed in the related literature [28], but the vibration mode of the sensing structure will change under the presence of thermal stress. The most typical effect is the frequency drift of the structure. The resonance frequency of the drive mode will drift when there is thermal stress inside the sensing structure of the BFVG. The resonance frequency of the drive mode of the equivalent double-clamped beam has previously been analyzed [29]. The formula can be expressed as

(1) $$\omega_{d}=\sqrt{\frac{k_{d}}{I_{d}}}=\sqrt{\frac{2E_{si}I_{y^{\prime }}}{I_{d}l}}$$

In the above formula, $k_{d}$ is the flexural stiffness of beam. $I_{d}$ is the moment of inertia of the drive mode. $E_{si}$ is the young modulus of silicon. $I_{y^{\prime }}$ is the principal moment of inertia of the cross-section of the oblique beam. l is the length of the beam.

The formula of resonance frequency of the sense mode can be expressed as

(2) $$\omega_{s}=\sqrt{\frac{k_{s}}{I_{s}}}=\sqrt{\frac{2G_{si}I_{p}}{I_{s}l}}$$

In the above formula, $k_{s}$ is the torsional stiffness of beam. $I_{s}$ is the moment of inertia of the sense mode. $G_{si}$ is the shear modulus of silicon. $I_{p}$ is the polar moment of inertia of the cross-section of the oblique beam.

The drive mode is the second vibration mode of the oblique beam. The sense mode is the third vibration mode of the oblique beam. When the axial thermal stress exists, the difference between their operating frequencies is [30]

(3) $$\begin{array}{cc} \Delta \omega & =\omega_{s}(1+\frac{\gamma_{s}Nl^{2}}{24G_{si}I_{s}})-\omega_{d}(1+\frac{\gamma_{d}Nl^{2}}{24E_{si}I_{s}})\\ & =\omega_{s}-\omega_{d}+(\frac{\gamma_{s}}{I_{s}}\omega_{s}\frac{Nl^{2}}{24G_{si}}-\frac{\gamma_{d}}{I_{s}}\omega_{d}\frac{Nl^{2}}{24E_{si}}) \end{array}$$

N represents the axial thermal stress of the sensitive structure. $\gamma_{d}$ and $\gamma_{s}$ represent the influence factor of the axial thermal stress on the vibration of the drive mode and the sense mode of the oblique beam, respectively. 

From the above formulas, it can be seen that the frequency mismatch between the operating modes changes with the axial thermal stress, and shows a fixed linear relationship. Therefore, the structural thermal stress can cause a prominent shift in the resonant frequency of the BFVG, and change the difference between the operating frequencies. 

The difference between the operating frequencies has a significant effect on the mechanical sensitivity of the BFVG. The mechanical sensitivity can be expressed as

(4) $$S=\frac{\Delta C_{S(\phi_{S0})}}{\Omega }=k_{\Delta C_{S}(\phi_{S0})}\frac{\sin\theta_{p}}{d^{2}}\frac{\phi_{d0}\omega_{d}}{\sqrt{{\left(\omega_{s}^{2}-\omega_{d}^{2}\right)}^{2}+{\left(\frac{\omega_{d}\omega_{s}}{Q_{S}}\right)}^{2}}}$$

where $\omega_{d}$ is the resonant frequency of the drive mode, $\omega_{s}$ is the resonant frequency of the sense mode, $Q_{s}$ is the related quality factor of the sense mode, Ω is the angular rate input, $\phi_{d0}$ is the vibration amplitude of the drive mode, $\theta_{p}$ is the spindle azimuth angle of the oblique beam $k_{\Delta C_{s(\phi_{s0})}}$ is the coefficient of the sensitivity equation.

In order to reflect the relationship between the mechanical sensitivity and the operating frequency mismatch, the vibration amplitude of the drive mode is assumed to be a constant, and the size parameters and quality factors of the BFVG remain unchanged. According to Equation (5), the sensitivity equation can be simplified as

(5) $$S=K*\frac{\omega_{d}}{{\left(\omega_{d}+\Delta \omega \right)}^{2}\sqrt{{\left(1-{\left(\frac{\omega_{d}}{\omega_{d}+\Delta \omega }\right)}^{2}\right)}^{2}+{\left(\frac{\omega_{d}}{(\omega_{d}+\Delta \omega )Q_{S}}\right)}^{2}}}$$

**Figure 4:**
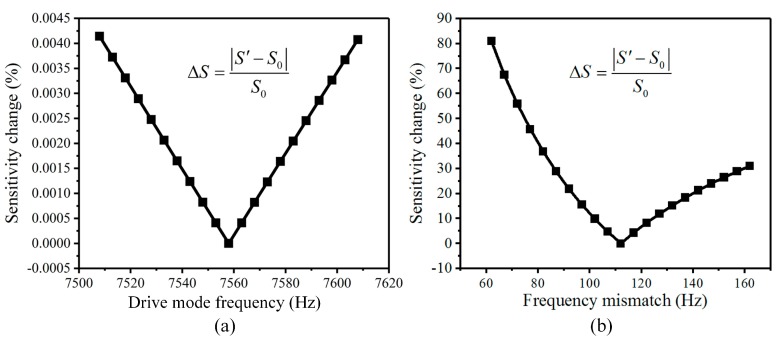
The sensitivity of the BFVG affected by the drive mode frequency and frequency mismatch: (**a**) The drive mode frequency; (**b**) the frequency mismatch.

Therefore, the value of the drive mode frequency of the BFVG is designed as 7557 Hz, the sense mode as 7670 Hz, and the frequency mismatch as 112 Hz. Under the condition that the other structural parameters are fixed, the single-variable method is adopted to analyze the mechanical sensitivity of the BFVG, which is influenced by the drive mode frequency and the frequency mismatch. First, the frequency mismatch is assumed to be constant. The change of the mechanical sensitivity is shown in Figure 4a, and the drive mode frequency ranges from 7508 Hz to 7608 Hz. Then, the drive mode frequency is assumed to be a constant. The frequency mismatch ranges from 62 Hz to 162 Hz. The influence on the mechanical sensitivity is shown in Figure 4b.

According to the previous results, the fluctuation of the frequency mismatch of the BFVG has a greater impact on the mechanical sensitivity than the drive mode frequency fluctuation. Therefore, minimizing the fluctuation of the frequency mismatch is the most important for improving the stability of the BFVG while maintaining the stability of the drive mode frequency.

## 3. The Optimization of the Stress-Released Structure of the BFVG

**Figure 5:**
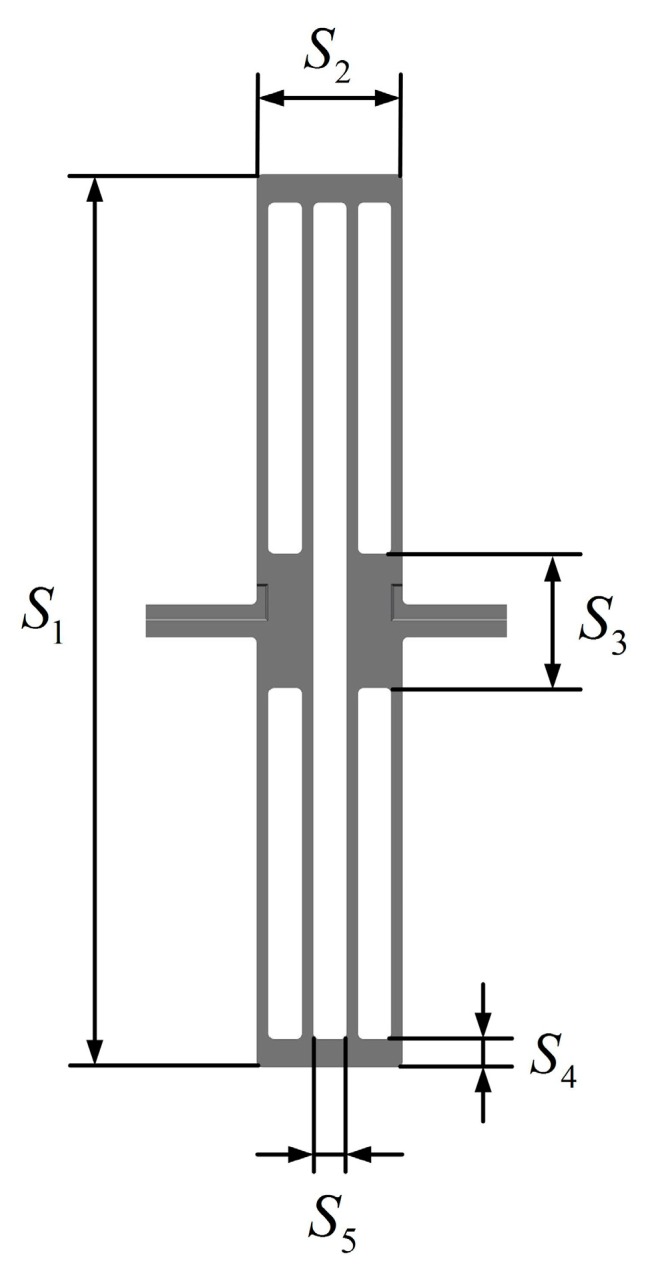
The main structural parameters of the stress-released structure of the BFVG.

The dimensional parameters of the stress-released structure relate to the effect of thermal stress release and the stiffness characteristics of the oblique beam. Therefore, it is very important to optimize the design of the stress-released structure. By analyzing the folded beam of the stress-released structure, the main structural parameters are shown in Figure 5. The parameters are as follows: the height of the stress-released structure (S_1_), the width of the stress-released structure (S_2_), the height of the internal fixed frame of the stress-released structure (S_3_), the height of the outer fixed frame of the stress-released structure (S_4_), and the width of the stress-release groove (S_5_).

The single parameter analysis method is used to simulate the temperature characteristics of the operating frequency and the frequency mismatch of the BFVG in the whole temperature range. The maximum variation of the drive mode frequency, the Sense mode frequency, and the frequency difference are obtained respectively. The influence of the parameters on the effect of thermal stress release is explored to guide the optimal size of the final design. 

### 3.1. Optimization of Stress-Released Structural Parameters of the BFVG

**Figure 6:**
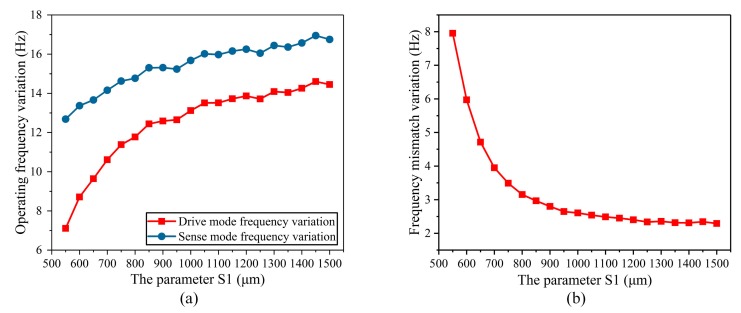
The parameter of the height of the stress-released structure (S_1_) affected the variation of the operating frequency and frequency mismatch: (**a**) The degree of variation of the operating frequency; (**b**) the degree of variation of the frequency mismatch.

Firstly, the height of the stress-released structure (S_1_) was analyzed. S_1_ ranges from 550 μm to 1500 μm. The temperature characteristics of the operating frequency and the frequency mismatch of the BFVG in the whole temperature area were simulated respectively in different sizes. Figure 6a shows the total variation of the frequency in the whole temperature range under different S_1_ parameters. The transverse coordinate of the figure is the range of parameter S_1_, and the vertical coordinate is the maximum variation of the frequency of the gyroscope’s drive and sense modes in the full range of absolute temperatures from 230 K to 340 K under the specified parameters S_1_. The red curve is the change of the drive mode frequency, and the blue curve is the change of the sense mode frequency. The analysis shows that the total change in the whole temperature range of the operating frequency increases with the increase of stress-released structure height. Figure 6b shows the total variation of the frequency mismatch in the whole temperature range under different S_1_ parameters. The transverse coordinate of the figure is the range of parameter S_1_, and the vertical coordinate is the maximum variation of the frequency mismatch of the gyroscope in the full range of absolute temperatures from 230 K to 340 K under the specified parameters S_1_. The total variation of the frequency mismatch decreases, rapidly, when the height of the stress-released structure ranges from 550 μm to 750 μm. After that, when S_1_ increases, the frequency mismatch variation is basically stable.

**Figure 7:**
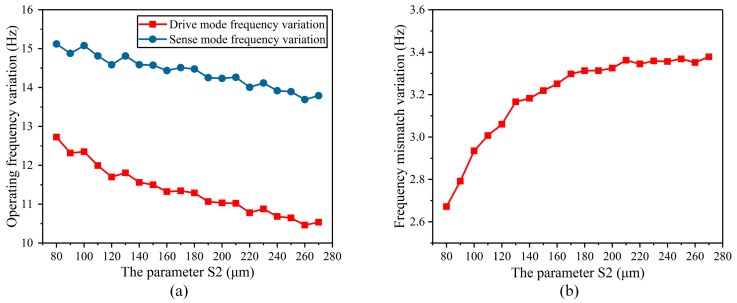
The parameter of the width of the stress-released structure (S_2_) affected the variation of the operating frequency and frequency mismatch: (**a**) The degree of variation of the operating frequency; (**b**) the degree of variation of the frequency mismatch.

Next, the width of the stress-released structure (S_2_) was analyzed. S_2_ ranges from 80 μm to 270 μm. The operating frequency variation decreases as S_2_ increases (Figure 7a). As S_2_ increases, the frequency mismatch variation becomes larger and more stable (Figure 7b). In addition, S_2_ has great influence on the width size of the BFVG, which needs to be considered comprehensively.

**Figure 8:**
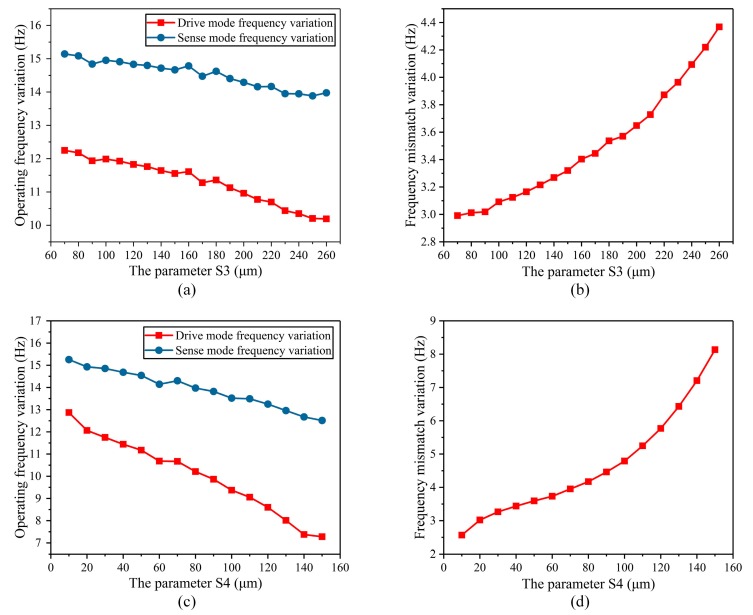
The parameter of the height of the internal fixed frame of the stress-released structure (S_3_) and the height of the outer fixed frame of the stress-released structure (S_4_) affected the variation of the operating frequency and frequency mismatch: (**a**) The degree of variation of the operating frequency of the parameter of S_3_; (**b**) the degree of variation of the frequency mismatch of the parameter of S_3_; (**c**) the degree of variation of the operating frequency of the parameter of S_4_; (**d**) the degree of variation of the frequency mismatch of the parameter of S_4_.

The parameters of the height of the internal fixed frame of the stress-released structure (S_3_) and the height of the outer fixed frame of the stress-released structure (S_4_) were analyzed. These two parameters mainly determine the height of the stress-release groove, so they have been analyzed together. As the height of the internal fixed frame of the stress-released structure needed to be wider than the gyroscope’s oblique beam, the dimension range of S_3_ was selected from 70 μm to 260 μm. The height of the outer fixed frame of the stress-released structure (S_4_) had no special requirements, so the dimension range of S_4_ was selected from 10 μm to 150 μm. The temperature characteristics of the operating frequency and the frequency mismatch of the BFVG in the whole temperature area were simulated respectively in different sizes. The simulation results are shown in Figure 8. The simulation results show that when S_3_ and S_4_ increases, the variation of the operating frequency in the whole temperature range decreases and the variation of the frequency mismatch increases. As can be seen from the above analysis, the variation of the frequency mismatch has a more significant impact on the BFVG’s sensitivity. Therefore, under the comprehensive consideration of the degree of the variation of the BFVG’s operating frequency, the size parameters with a small variation of the frequency mismatch are selected.

**Figure 9:**
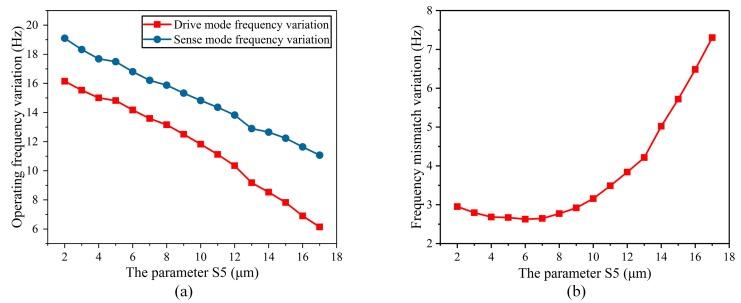
The parameter of the width of the stress-release groove (S_5_) affected the variation of the operating frequency and frequency mismatch: (**a**) The degree of variation of the operating frequency; (**b**) the degree of variation of the frequency mismatch.

At last, the width of the stress-release groove (S_5_) was analyzed. It was closely related to the width of the stress-released structure (S_2_). Therefore, after the optimization design of width of the stress-released structure, the temperature characteristics of the operating frequency and frequency mismatch of the BFVG under different S_5_ were analyzed. According to the above analysis, S_2_ was selected to be 130 μm, so S_5_ ranged from 2 μm to 17 μm. Figure 9a shows the variation of the operating frequency in the whole temperature range under different S_5_ parameters. It can be seen that the degree of variation of the operating frequency decreases as S_5_ increases. Accordingly, at a larger value of S_5_, the variation of the frequency mismatch increases as does its rate of change with respect to S_5_. Accordingly, the variation of the frequency mismatch is increasing and the rate is getting more rapidly.

According to the theoretical analysis of the above section, the influence of frequency drift of the frequency mismatch on the sensitivity of the BFVG is much greater than that of the drift of the operating frequency. Therefore, on the premise that the drive mode frequency of the BFVG is stable, the drift of the frequency mismatch caused by thermal stress should be reduced to the maximum extent, which can significantly improve the performance of the BFVG. It is a greatly significant way to improve the stability of the BFVG. The simulation data show that the main parameters affecting the effect of thermal stress-released structure include the height of the stress-released structure (S_1_), the height of the outer fixed frame of the stress-released structure (S_4_), and the width of the stress-release groove (S_5_). In addition, the gyroscope’s frequency mismatch caused by the change of the width of the stress-released structure (S_2_) and the height of the internal fixed frame of the stress-released structure (S_3_) of the stress-released structure is relatively stable, while the dimension of the width of the stress-released structure (S_2_) relates to the size of the gyroscope as a whole. To sum up, various parameters of the stress-released structure of the BFVG were reasonably designed, and the final optimized dimensions were obtained, as shown in Table 1.

### 3.2. Thermal Stress Analysis of the BFVG

The structural thermal stress causes a significant drift in the BFVG’s resonant frequency, which can lead to a change of the frequency mismatch. The change of the frequency mismatch has a distinct effect on mechanical sensitivity. Therefore, minimizing the fluctuation of the frequency mismatch can effectively improve the stability of the BFVG. In order to verify the effectiveness of the new stress-released structure, the BFVG with new stress-released structure and the BFVG with no stress-released structure are simulated and compared with each other in the full range of absolute temperatures from 230 K to 340 K by COMSOL software 5.2 (Stockholm, Sweden). In order to reduce the structural deformation caused by the different thermal expansion coefficient of the different materials, the BFVG in this paper is made of all-silicon material. The material properties of the silicon set in the COMSOL software are shown in Table 2.

**Figure 10:**
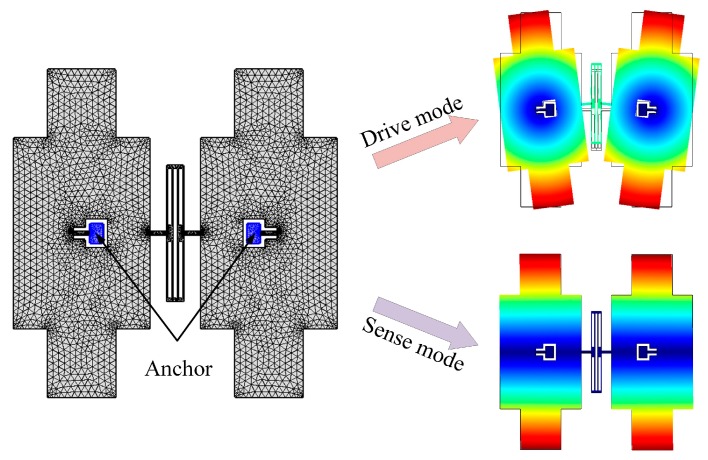
The simulation condition in the COMSOL software.

Taking the BFVG with new stress-released structure model as an example, after meshing in the COMSOL software, the symmetry distribution anchors are fixed as shown in Figure 10. The frequency parameterized scanning is carried out in the full range of absolute temperatures from 230 K to 340 K. The drift degree of the frequencies of the drive and sense modes by temperature change are obtained respectively.

**Figure 11:**
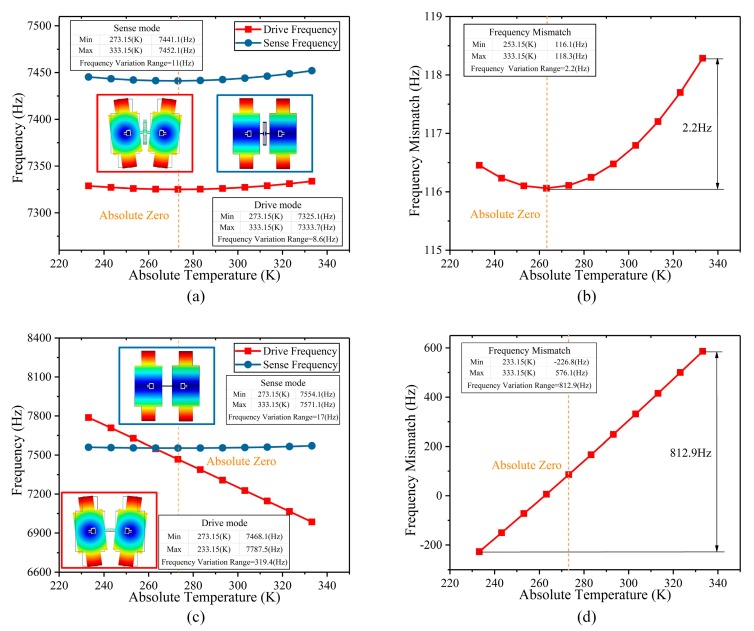
The changes of the operating frequency and frequency mismatch of the BFVG with or without the new stress-released structure (**a**) The variation of the operating frequency with the new stress-released structure; (**b**) the variation of the frequency mismatch with the new stress-released structure; (**c**) the variation of the operating frequency without the new stress-released structure; (**d**) the variation of the frequency mismatch without the new stress-released structure.

The changes in the operating frequency and the frequency mismatch of the BFVG with or without the new stress-released structure are shown in Figure 11. From the simulation results of the BFVG with the new stress-released structure, the drive mode frequency changes by 8.6 Hz, the Sense mode frequency changes by 11 Hz, and the frequency mismatch changes by 2.2 Hz. Furthermore, from the simulation results of the BFVG without the new stress-released structure, the drive mode frequency changes by 319.4 Hz, the Sense mode frequency changes by 17 Hz, and the frequency mismatch changes by 812.9 Hz. According to the results, under different working temperatures, the BFVG with the new stress-released structure has a significantly reduced degree of change in its operating frequencies and frequency mismatch. As a result, this new stress-released structure achieves the purpose of structural optimization. It has a great effect of removing the thermal stress. 

## 4. The Performance Test of the BFVG

**Figure 12:**
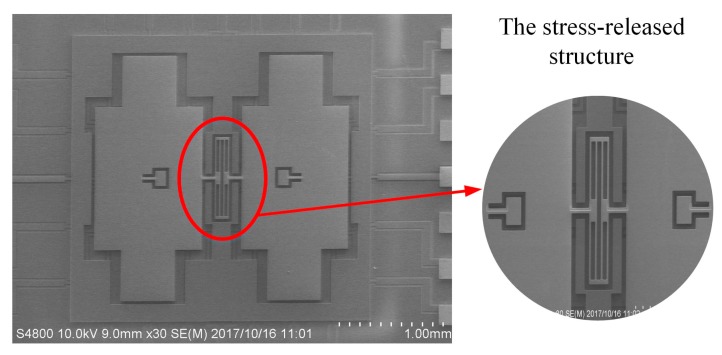
The electron microscope photos of the sensing structure of the BFVG.

In order to reduce the structural thermal stress caused by the mismatch of material properties, this BFVG is made using all-silicon material. Through the reasonable fabrication processes, the BFVG with the new stress-released structure has been manufactured. The electron microscope of the BFVG is shown in Figure 12 (the package cap is removed). The machined dimensions of the stress-released structure of the BFVG are measured. The width of the stress-released beams is identical. After that, the performance of the BFVG will be tested, including the thermal stress, the quadrature trimming, and the Allan deviation. The experimental results show that the optimum design of the BFVG can effectively improve the performance.

**Figure 13:**
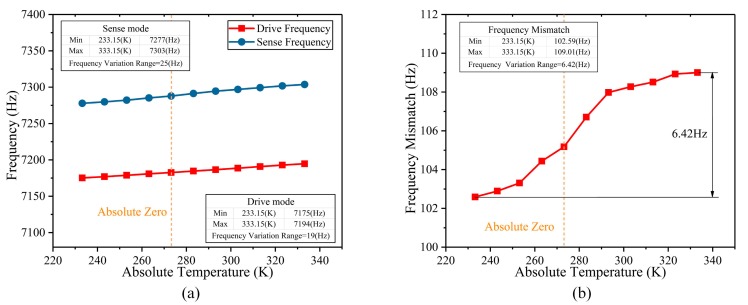
The actual testing changes of the operating frequency and frequency mismatch of the BFVG with the new stress-released structure (**a**) The variation of the operating frequency; (**b**) the variation of the frequency mismatch.

The experiment of the frequency change caused by thermal stress in the temperature oven chamber is carried out. The BFVG with its control circuit is put in the oven chamber to evaluate the thermal stress release of its operating frequency and frequency mismatch drift. The temperature range of the oven chamber is configured from 230 K to 340 K. The changes of the operating frequency and frequency mismatch of the BFVG with the new stress-released structure are shown in Figure 13. Based on the results of the actual testing, under different working temperatures, the drifting trend of operating frequency and frequency mismatch of the BFVG are similar to the simulation results. It shows that this new stress-released structure can reduce the thermal stress and improve the performance of the BFVG.

## 5. Conclusions

In this paper, a new architecture of the Butterfly Vibratory Gyroscope with better symmetry is developed. The new stress-released structure can reduce the effect of thermal stress to improve the BFVG’s performance. Due to the material properties and the fabrication processes, the deformation caused by residual stress or thermal mechanical stress is of great concern since it directly affects the performance in many gyroscopes (including the BFVG). Through the analysis of theoretical foundations of thermal stress, the induced mechanical deformation and the thermal mechanical stress have been confirmed to directly affect the frequency mismatch of the BFVG, and it will influence the performance of the BFVG. In order to improve the performance, this deformation should be controlled or eliminated. Therefore, the new stress-released structure is designed, considering the symmetry of the electrode and the miniaturization of the structure, to reduce the effect of thermal stress on gyroscope’s performance. In order to optimize the size parameters of the new stress-released structure, the single parameter analysis method is used to simulate the temperature characteristics of the operating frequency and the frequency mismatch of the BFVG in the whole temperature range to guide the final design. By comparing the theoretical and experimental results, this optimum design is verified that can effectively reduce the influence of thermal stress on the gyroscope’s performance.

## Figures and Tables

**Table 1:** Structural parameters of the stress-released structure (unit: μm).

Definition	Symbol	Value
the height of the stress-released structure	S_1_	1000
the width of the stress-released structure	S_2_	130
the height of the internal fixed frame of the stress-released structure	S_3_	120
the outer fixed frame of the stress-released structure	S_4_	25
the width of the stress-release groove	S_5_	10

**Table 2:** The material properties of the silicon.

Definition	Value	Unit
Thermal Expansion Coefficient	2.6 × 10^−6^	1/K
Density	2329	Kg/m^3^
Young Modulus	170 × 10^9^	Pa
Poisson Ratio	0.28	-
